# The Isopropylstilbene Precursor Cinnamic Acid Inhibits Anthraquinone Pigment Production by Targeting AntI

**DOI:** 10.1021/jacs.5c07388

**Published:** 2025-06-06

**Authors:** Li Su, Maximilian Schmalhofer, Gina L. C. Grammbitter, Nicole Paczia, Timo Glatter, Michael Groll, Helge B. Bode

**Affiliations:** † Department of Natural Products in Organismic Interactions, Max Planck Institute for Terrestrial Microbiology, 35043 Marburg, Germany; ‡ Center for Protein Assemblies, Department of Bioscience, TUM School of Natural Sciences, Technical University of Munich, 85748 Garching, Germany; § Molecular Biotechnology, Department of Biosciences, 9173 Goethe University Frankfurt, 60438 Frankfurt am Main, Germany; ∥ Core Facility for Metabolomics and Small Molecule Mass Spectrometry, Max Planck Institute for Terrestrial Microbiology, 35043 Marburg, Germany; ⊥ Core Facility for Mass Spectrometry & Proteomics, Max Planck Institute for Terrestrial Microbiology, 35043 Marburg, Germany; # Department of Chemistry, Philipps University of Marburg, 35043 Marburg, Germany; ⊗ Center for Synthetic Microbiology (SYNMIKRO), Philipps University of Marburg, 35043 Marburg, Germany

## Abstract

*Photorhabdus* strains, Gram-negative bacteria pathogenic to insect larvae, produce two signature compounds: the multifunctional isopropylstilbene (IPS), known for its antibiotic, insecticidal, and immunosuppressive activities, and orange-to-red pigmented anthraquinones (AQs), which attenuate oxidative stress. Here, we demonstrate an inverse correlation between the production of AQs and cinnamic acid (CA), the primary precursor for IPS formation in the model strain *P. laumondii* TTO1. Metabolic and proteomic analyses following CA treatment show that CA inhibits AntI, a key enzyme in the final step of AQ-256 biosynthesis. The crystal structure of AntI in complex with CA reveals that cinnamic acid functions as a competitive inhibitor by inducing specific structural rearrangements in the lyase, resulting in noncovalent, reversible inhibition. These findings provide atomic insights into the intricate regulatory control of pigment biosynthesis and the production of bioactive compounds.

## Graphical abstract

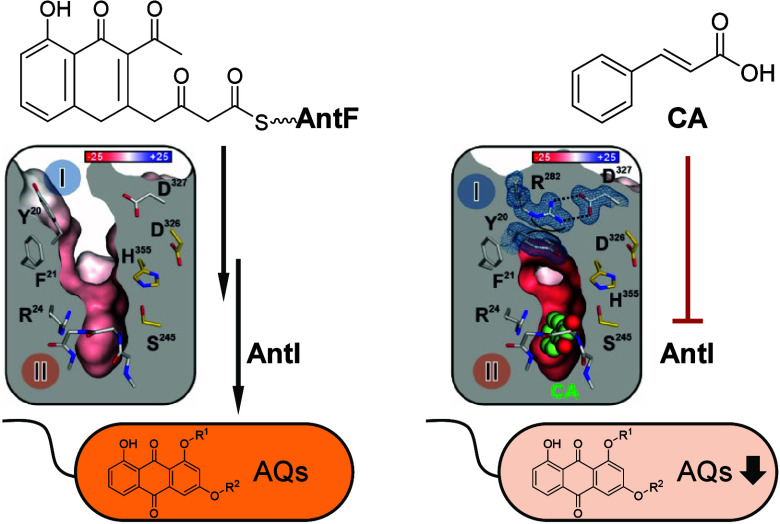

*Photorhabdus* spp. are Gram-negative bacteria that live in mutualistic symbiosis with *Heterorhabditis* nematodes and are pathogenic to insect larvae.^1^ These entomopathogenic bacteria possess a specialized metabolism that produces a wide range of compounds, including antibiotics, bioluminescent agents and pigments.^1–5^ Some of these molecules are crucial for symbiosis, while others are involved in quorum sensing, protection of insect carcass from predators, or oxidative stress response.^1,6^

Here we analyzed the endogenous regulation of anthraquinone (AQ) pigments, which give *Photorhabdus* species their characteristic orange-to-red coloration during exponential growth.^7,8^ AQs are aromatic polyketides produced by a type II polyketide synthase system (PKS), which is rarely found in Gram-negative bacteria.^8^ The corresponding gene cluster (*antA*–*J*) in *P. laumondii* TTO1 ^9^ has been studied recently and encodes a series of enzymes including the ‘minimal PKS’ system composed of ketosynthase (KS_α_), chain length factor (CLF/KS_β_), and acyl carrier protein (ACP) (Figure S1).^7,10,11^ In the minimal PKS associated with AQ synthesis, the starter unit malonyl-CoA is transferred to the ACP (AntF) via malonyl-CoA:ACP transacylase (MCAT) encoded outside of the *ant* gene cluster, followed by seven rounds of iterative decarboxylative condensation of malonyl-ACP via the AntD:E (KS_α_-KS_β_) complex, resulting in the formation of the octaketide linked to AntF^10,11^ (Figure 1a, Figure S1). After partial cyclization, the AntF-bound intermediate is further processed to a heptaketide by the unusual chain length shortening lyase AntI.^10,12^ Spontaneous oxidation of precursor compounds results in the red pigment AQ**-256**, which exhibits a tricyclic structure. Finally, a series of *O*-methyltransferases (MTs) encoded outside the *ant* gene cluster introduce one or two methyl groups to AQ**-256**, producing its mono- and dimethylated derivatives, AQ**-270** and AQ**-284**, respectively.^13^ These anthraquinones, particularly AQ**-270a** and AQ**-284a**, are the predominant AQs observed in TTO1 (Figure 1a, Figure S1, and Figure 2).

**Figure 1:**
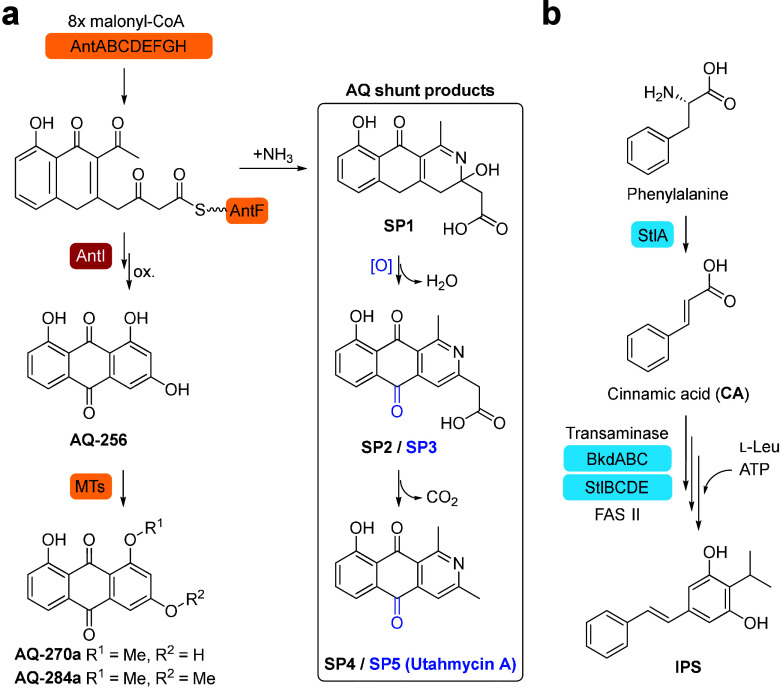
Biosynthesis pathway of (a) anthraquinone (AQ) and its shunt products (SP1–5), and (b) isopropylstilbene (IPS).

**Figure 2:**
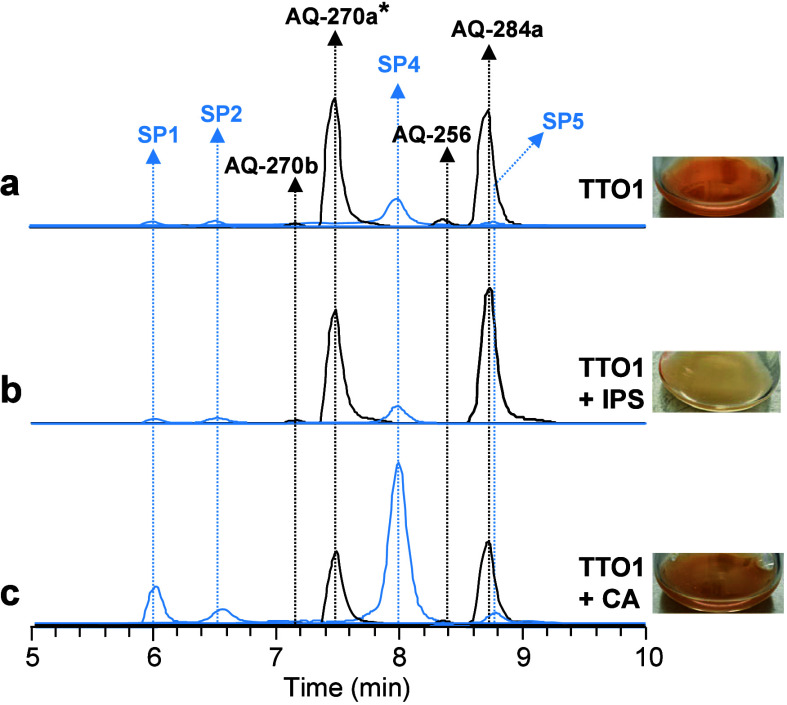
Extracted ion chromatograms (EICs) of AQs and AQ shunt products (SPs) from wild-type TTO1 in different culture conditions after 2 days. Asterisk means peaks of AQ-270a were 5-fold decreased in order to fit it into the chromatogram.

Notably, isopropylstilbene (IPS) is a key metabolite of TTO1, and modulates the transcription of several genes involved in pigment production including AQs.^14–16^ IPS biosynthesis starts with the deamination of phenylalanine to cinnamic acid (CA), catalyzed by stilbene synthase A (StlA, Figure 1b and Figure S2). Deletion of the *stlA* gene in TTO1 (Δ*stlA*) induces hyperpigmentation,^14,17,18^ highlighting the crucial role of CA and IPS in pigment formation. Exogenous addition of IPS in a concentration of 126 μM can significantly cause color bleaching in wild-type TTO1 (Figure 2b),^16^ whereas higher concentrations exceeding 0.5 mM are associated with cytotoxic effects.^15^ In contrast to IPS, the exogenous addition of 1.3 mM CA to TTO1 did not show any negative effect on cell growth because the majority of CA is metabolized to the tricarboxylic acid cycle intermediates, whereas only a small fraction of CA is converted to IPS due to the tight regulation of IPS biosynthesis.^19^ Interestingly, we have observed that the addition of 1 mM CA also resulted in visible change in pigmentation of the wild-type TTO1 strain (Figure 2c).

To investigate whether IPS- and CA-induced color reduction is linked to AQ biosynthesis, we compared AQ-**256**, AQ-**270a**, and AQ-**284a** levels using high-resolution liquid chromatography–mass spectrometry (HR-LCMS, Table S1). In TTO1, IPS addition led to a slight decrease in AQ-**270a**, while AQ-**284a** levels increased (Figure 2b, Figure S3). In contrast, CA addition significantly reduced both AQ-**270a** and AQ-**284a** (Figure 2c, Figure 3a, and Figure S4a). Similar trends were observed in the Δ*stlA* mutant, where IPS and CA induced comparable changes in culture color and AQ production (Figure 3b, Figure S4b, and Figure S5). Thus, our findings indicate that CA-induced color changes correlate with reduced AQ biosynthesis, whereas IPS primarily causes color bleaching without altering overall AQ levels.

**Figure 3:**
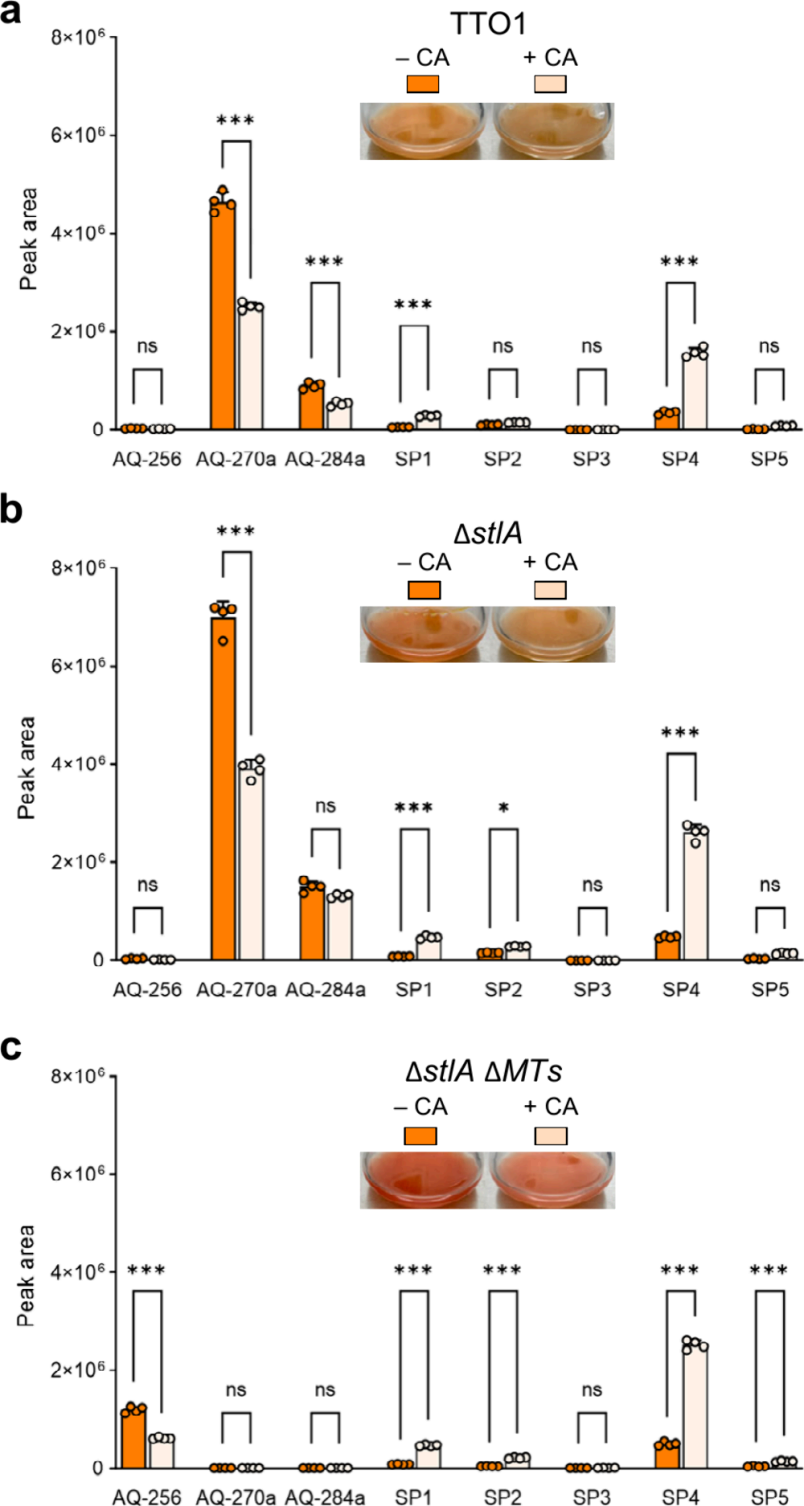
HR-LCMS analysis of the production of AQs and AQ shunt products SP1-SP5 in the absence/presence of fed CA after 2 days. Error bars indicate mean ± s.d. for four replicate samples. Statistical analysis was performed with the Bonferroni’s multiple comparisons test. Asterisk show significant difference between CA-fed and non CA-fed samples (ns = not significant, * = *P* ≤ 0.033, **= *P* ≤ 0.002, *** = *P* ≤ 0.001.). Figure S4 shows the same analysis after 1 day.

To further elucidate the role of CA in AQ biosynthesis, we constructed a new strain, Δ*stlA* Δ*MTs*, by deleting the *O*-methyltransferase genes *plu4890*, *plu4891*, *plu4892*, *plu4894*, and *plu4895* (Figure S1) in the Δ*stlA* variant.^13^ These genes are responsible for the conversion of AQ-**256** to AQ-**270a** and AQ-**284a**. Therefore, the Δ*stlA* Δ*MTs* mutant prevents postmethylation modifications, allowing us to assess whether CA specifically regulates AQ-**256** formation. Indeed, upon addition of CA, we observed both culture discoloration and a significant decrease in AQ-**256** levels (Figure 3c, Figure S4c). To identify the precise target of CA, we analyzed shunt products (SPs) generated due to impaired AQ-**256** biosynthesis. Following CA treatment, Δ*stlA* and Δ*stlA* Δ*MTs* exhibited a drastic accumulation of SPs, particularly **SP1**, **SP2**, and **SP4** (Figure 3b, c), whereas no changes were detected after IPS treatment (Figure 2b, Figure S5). Interestingly, these SPs arise from the assimilation of intracellular NH_3_ into AntF-bound octaketide (Figure 1) and were previously identified in the TTO1 Δ*antI* mutant,^10^ indicating that CA blocks polyketide shortening by AntI. To determine whether CA affects the expression of AQ biosynthesis genes, we conducted proteomic analyses of TTO1, Δ*stlA*, and Δ*stlA* Δ*MTs* in the presence and absence of CA. Unlike IPS, which was previously reported to downregulate AQ biosynthetic genes^16^ and did indeed not result in a significant upregulation of the AQ biosynthesis enzymes (Figure S6), CA treatment led to a significant upregulation of AntI in all three strains (Figure 4). Taken together, these findings suggest that the increased AntI production following CA treatment likely serves as a compensatory response to CA-induced AntI inhibition.

**Figure 4:**
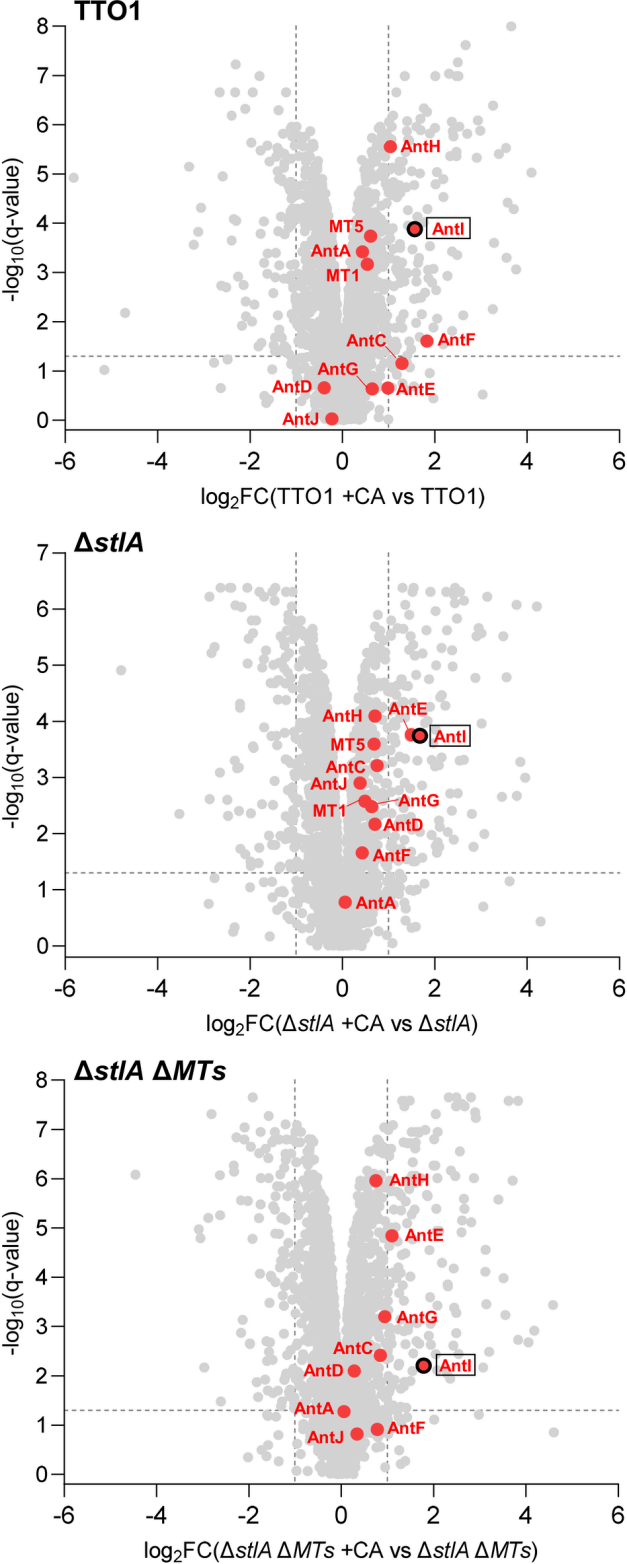
Volcano plot of the comparative proteomic analysis for TTO1, Δ*stlA*, and Δ*stlA* Δ*MTs* in the presence and absence of CA. Red dots indicate the proteins involved in AQ biosynthesis, AntI is highlighted with black circle. Dashed lines show cutoff values *q* = 0.05, and FC (fold change) = 2.

To elucidate the molecular mechanism of AntI inhibition by cinnamic acid, we solved the AntI:CA complex structure at 1.4 Å resolution (PDB ID 9GLF, Figure 5a). The analysis revealed that CA functions as a noncovalent inhibitor of AntI. The complex forms a homodimer with a single subunit in the asymmetric unit, and CA is well-defined in the F_o_-F_c_ electron density map and occupies the substrate binding channel. Structural comparisons with ligand-free AntI show that in the presence of CA, both the inhibitor and the active site remain compartmentalized in the closed state. In the open state, the Pro281-Arg283 loop (region I) and Tyr20 are exposed to solvent. However, upon CA binding, this segment undergoes a 90° rotation, causing the side chain of Arg282 to flip inward, while Tyr20 rotates by 120° (Figure 5b). These structural rearrangements facilitate π-stacking between Arg282 and Tyr20, leading to the formation of two strong salt bridges between Arg282 and Asp327 (Figure 5c). Notably, the interactions involving Tyr20 and Arg282 are further stabilized by an acetate buffer molecule, a feature previously observed in the closed state of AntI.^12^ Although the tight aromatic network extending to the styrene moiety of CA favors this configuration, no pronounced interactions are observed between these residues and the inhibitor itself. In summary, while the open-to-closed transition of AntI is essential for catalytic activity, CA binding alone does not appear to induce active site closure, which likely occurs after the retro-Claisen reaction.^12^ Instead, CA acts as a competitive inhibitor by occupying the substrate-binding channel.

**Figure 5:**
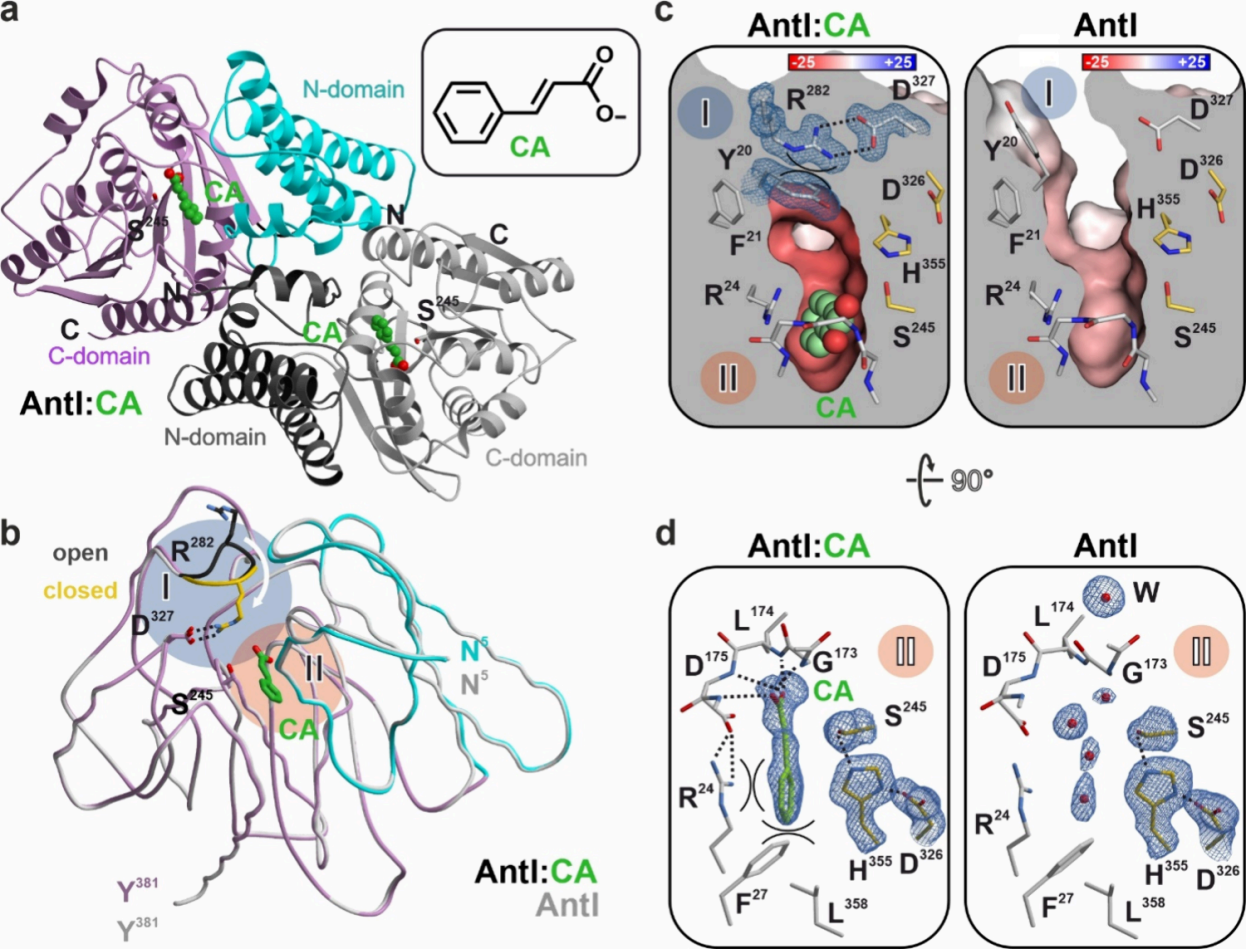
Binding mode of CA to AntI. a) Ribbon representation of dimeric AntI in complex with the inhibitor CA (carbon atoms in green; PDB ID 9GLF). b) The superposition of AntI^apo^ (gray, PDB ID 6HXA,^10^ open state) with AntI:CA complex structure (closed state) shows the inhibitor bound in a central cavity (region II). The loop connecting residues Pro281-Arg283 is rotated by 90° from black (AntI^apo^) to gold (AntI:CA). c) Sliced surface representations of the closed (ligand bound) and open states of AntI with electrostatic potentials. d) Atomic view of the active site in the CA-bound complex (left panel) and ligand-free open state structure (right panel). Yellow sticks represent the aligned catalytic triad, while surrounding residues are shown in gray. Water molecules are depicted as red spheres, with H-bonds indicated by dashed lines and π-stacking interactions by semicircles. CA is shown in green. The Fo-Fc electron density map for CA and the active site residues is displayed as blue mesh and contoured at 3.0 σ.

In the AntI:CA complex, the catalytic triad remains perfectly aligned (region II), with the active site nucleophile Ser245 positioned just 3.4 Å from the styrene moiety of CA. However, the conjugated π-electron system of the aromatic ring stabilizes the double bond, preventing Ser245 from forming a covalent bond with the ligand. This explains the reversible mode of CA inhibition (Figure 5d, left panel). Although CA binding is entropically favored by the displacement of mobile water molecules from the specificity pocket (Figure 5d, right panel), its enthalpic interactions with AntI are particularly strong. The styrene residue of CA engages in cation-π stacking with Arg24 and π-π stacking with Phe27, while its carboxylate group forms hydrogen bonds with the backbone amides of Gly173, Leu174, and Asp175. A structural comparison of the AntI:CA complex with ligand-free AntI in its open and closed states^12^ reveals that all surrounding residues retain similar orientations, except for Gly173. In the ligand-free state, Gly173 forms a β-turn, coordinating a well-defined water molecule (W) at 2.8 Å. However, in the CA-bound structure, the carboxylate group of CA displaces Gly173 and induces a 37° rotation of its carbonyl group (Figure 5d). This structural rearrangement imposes geometric constraints that generate energetically unfavorable φ- and ψ-dihedral angles in the protein backbone within region II, counteracting the otherwise favorable ligand interactions at the active site. Thus, our structural findings demonstrate that CA functions as an endogenous, reversible, and competitive inhibitor of AntI, preventing its catalytic activity. By occupying the substrate-binding channel, CA effectively suppresses shunt product formation while preserving the integrity of the AntF-substrate complex, thereby playing a key regulatory role in AQ biosynthesis.

In conclusion, *Photorhabdus laumondii* TTO1 exhibits a phenotypic response to its own metabolite, CA, through direct inhibition of AntI, a key enzyme in AQ-256 biosynthesis. In addition to the previously observed IPS-dependent transcriptional downregulation of pigments,^16^ here we uncover a novel regulatory mechanism in which CA directly interacts with AntI to control anthraquinone pigment biosynthesis. Furthermore, our study highlights the intricate metabolic interplay between these two signature molecules, providing new insights into the regulatory complexity of secondary metabolite production in *Photorhabdus*.

## Supplementary Material



## Notes

Experimental details, metabolomic data (HR-LCMS), proteomic data, structure data and refinement statistics of AntI:CA crystal complex (PDF)
