# Supplementary material for: The Isopropylstilbene Precursor Cinnamic Acid Inhibits Anthraquinone Pigment Production by Targeting AntI
Source: J Am Chem Soc. 2025 Jun 6;147(24):20246–50. doi: 10.1021/jacs.5c07388 (PMC12186476; doi:10.1021/jacs.5c07388)
Supplement: Supplementary file 1 [file ja5c07388_si_001.pdf]

Supplementary Information

The Isopropylstilbene Precursor Cinnamic Acid Inhibits Anthraquinone Pigment Production by Targeting AntI

Li Su[1,‡], Maximilian Schmalhofer[2,‡], Gina L. C. Grammbitter[3,‡], Nicole Paczia[4], Timo Glatter[5], Michael Groll[2,*], Helge B. Bode[1,3,6,7*]

[1] Department of Natural Products in Organismic Interactions, Max Planck Institute for Terrestrial Microbiology, 35043 Marburg, Germany

[2] Technical University of Munich, TUM School of Natural Sciences, Department of Bioscience, Center for Protein Assemblies, 85748 Garching, Germany

[3] Molecular Biotechnology, Department of Biosciences, Goethe University Frankfurt, 60438 Frankfurt am Main, Germany

[4] Core Facility for Metabolomics and Small Molecule Mass Spectrometry, Max Planck Institute for Terrestrial Microbiology, 35043 Marburg, Germany

[5] Core Facility for Mass Spectrometry & Proteomics, Max Planck Institute for Terrestrial Microbiology, 35043 Marburg, Germany

[6] Chemical Biology, Department of Chemistry, Philipps University of Marburg, 35043 Marburg, Germany

[7] Center for Synthetic Microbiology (SYNMIKRO), Philipps University of Marburg, 35043 Marburg, Germany

* Corresponding authors e-mail: michael.groll@tum.de, helge.bode@mpi-marburg.mpg.de

‡ These authors contributed equally.

Experimental Section

Culture and media. Unless otherwise specified, all strains were cultured in LB medium (10 g/L tryptone, 5 g/L yeast extract, and 5 g/L NaCl) or on an LB agar plate (1.5 % agar was added) at 37 ºC (for *E.coli* strains) or 28 ºC (for *Photorhabdus* strains). Antibiotic kanamycin (50 µg/mL) was added when appropriate. For the production culture of TTO1 and its mutants, the overnight LB culture was transferred into 5 mL LB medium with a starting OD_{600} of 0.1, followed by shaking at 28 ºC with 200 rpm for one and two days. In the feeding experiments, cinnamic acid (CA, Sigma-Aldrich, dissolve in ethanol) and IPS (Bode lab, dissolved in DMSO) were supplemented to the culture when OD_{600} around 0.45 in a final concentration of 1 mM (CA) or 126 µM (IPS). Cultures not supplemented with CA or IPS had an equal volume of ethanol or DMSO added.

HR-HPLC-MS analysis. After one and two days shaking, 10 µL of TTO1 and its mutant production cultures were taken, 90 µL MeOH was added and vortexed for 2 min, followed by centrifuged at 13,000 rpm for 20 min. The resulting samples were analyzed by HR-LCMS on Agilent Infinity II HPLC coupled to an Agilent 6550 Quadrupole Time-Of-Flight (Q-TOF) mass spectrometer using an ACQUITY UPLC BEH C18 column (130 Å, 2.1 mm × 50 mm, Waters) at a flow of 0.4 mL/min (5–95% acetonitrile/water with 0.1% formic acid, v/v, 20 min). Mass spectra were recorded in positive-ion-mode using the mass analyzer at the mass resolution of 40000. The EIC (extracted ion chromatogram) peak integrations were carried out automatically utilizing programs in the Agilent MassHunter Quantitative analysis (**Table S1**).

Construction of mutant Δ*stlA* Δ*MTs* by CRISPR/Cpf1. Strain Δ*MTs* was obtained from previous study[1] and was selected as the starting strain for the markerless deletion of Δ*stlA* using CRISPR/Cpf1 technique as described[2] Briefly, two crRNA sequences were selected to match the gene *stlA* which contains the protospacer adjacent motif (PAM) TTTV on its 5'-end (V is A, C, or G nucleotides) and a spacer sequence of 31 bp: TTTG(PAM)-GAAGATATCTATGACATTGCGATAAAACAAA(spacer A) and TTTA(PAM)-GCAGTTTCAG GCGCAATTTCACTAATATTGC (spacer B). Hereafter, the homology repair arms (HAL and HAR, each around 500 bp) were each coupled with one of the crRNAs and synthesized as a dsDNA fragment. The dsDNA fragments ΔstlA_L and ΔstlA_R were orderly assembled with pAR20 via Golden Gate Assembly (BsaI), resulting in pAR20_ΔstlA (**Table S3**). The pAR20_ΔstlA was then electro-transformed into *E. coli* ST18 and introduced to *Photorhabdus laumondii* TTO1 Δ*MTs* via conjugation. The resulting conjugants were firstly selected by their kanamycin resistance and then followed by addition of AHT (Anhydrotetracycline, 200 ng/mL final concentration) and arabinose (0.4% final concentration) to induce the λ-Red recombination and Cpf1 editing. Successful editing mutants were confirmed by colony PCR (LS144 + LS145, **Table S4**) and sequencing. Finally, the plasmid curing mutants were selected by sucrose (10% final concentration) to be the right Δ*stlA* Δ*MTs* mutant for further analysis.

Statistical analysis. Analysis of variance (2-way ANOVA) of the data was performed using GraphPad Prism 9.0.2 software. *P*-values were calculated by Bonferroni's multiple comparison test and are presented as: ns = not significant, * = P≤0.033, **= P≤0.002, *** = P≤0.001.

Proteomic analysis. A fresh 5 mL LB culture of TTO1, Δ*stlA,* and Δ*stlA* Δ*MTs* were inoculated from an overnight culture at OD_{600} of 0.1. CA and IPS were added to the culture when OD_{600} around 0.45 in a final concentration of 1 mM and 126 µM (equal to 32 µg/mL, the dosage used in a previous study[3]), respectively.

Cells were grown with shaking at 28 ºC until the OD_{600} of 2, 1.5 mL were harvested at 10000 rpm for 1 min. The cell pellets were washed twice with cold PBS buffer at 10000 rpm for 1 min. Afterward, the cell pellets were frozen in liquid nitrogen and stored at –80 ºC. Samples were prepared in biological quadruplicates. Procedures for sample processing and HPLC-MS/MS analysis were the same as previously described[4]. For the data presented in **Figure 4** and **Figure S6**, the threshold levels to assign the differentially expressed proteins between CA/IPS-supplemented and un-supplemented were relative fold change > 2 and the *q*-value < 0.05 based on samples with four biological replicates. Each point in the plot corresponds to the relative fold change between supplemented and un-supplemented for the peptides with minimal *q*-value among all peptides quantified for a certain protein, and thus one protein is represented by a single dot in the graph.

Protein expression and purification. Experiments were performed as previously described[5]. In brief, the recombinant protein was expressed overnight at 20 °C in *Escherichia coli* cells using lysogeny broth media and isopropylthiogalactoside for induction. Harvested cells were washed with 0.9 % (w/v) saline, thawed in 100 mM Tris/HCl, pH 8.0, containing 300 mM NaCl, 20 mM imidazole/HCl, 10 % (v/v) glycerol and 2 mM 2-mercaptoethanol (buffer A) and disrupted by sonification. For purification, the supernatant was applied onto a 5 ml HisTrap HP column (Cytivia, equilibrated and washed with buffer A) and eluted with buffer A containing 500 mM imidazole. After dialysis against 20 mM Tris pH 7.5, 100 mM NaCl, 2.5 mM dithiothreitol (buffer B), the protein was applied onto a HiLoad™ 16/600 Superdex™ 200 column (Cytivia, equilibrated and developed with buffer B). The purified protein was concentrated to a concentration of 25 mg/ml using a 30k Amicon Ultra Centrifugal Filter Device (Millipore), flash frozen in liquid nitrogen and stored at –80 °C.

Crystallization of AntI:CA. The protein (15 mg/mL) was co-crystallized with the inhibitor cinnamic acid (100 mM stock solution in DMSO:H_2O (1:1)) using the hanging drop vapor diffusion method at 20 °C. CA was added to AntI to final concentrations between 2 and 10 mM and incubated at room temperature for one hour or at 4 °C for 16 hours. Crystallization drops had a volume of 2 µl with a 1:1 ratio of protein to reservoir (0.1 M sodium acetate pH 6.6, 0.1 M (4-(2-hydroxyethyl)-1-piperazineethanesulfonic acid (pH range between 7.0 and 8.0) and 21-26 % polyethylene glycol 3350. Crystals were cryoprotected with 5 µl of a 1:1 mixture of mother liquor and 60% (v/v) ethylene glycol and vitrified in liquid nitrogen.

Structure determination of AntI:CA. Diffraction images of AntI:CA were recorded using synchrotron radiation of λ = 1.0 Å at the X06SA-beamline, Swiss Light Source (SLS), Paul Scherrer Institute, Villigen, Switzerland. The reflections were processed using the XDS suite[6] (**Table S5**) and the topology of the ligand was calculated with PRODRG[7]. Phases were obtained by Patterson Search calculations using coordinates of AntI^{apo} (PDB ID 6HXA)[5] and applying REFMAC5[8]. Restrained refinement of the structure with REFMAC5 was alternated by model building with COOT (v. 0.9)[9]. Water molecules were placed using ARP/wARP 8.0[10] and the overall geometry of the structure was analyzed by MOLPROBITY[11]. The crystal structure was deposited in the RCSB Protein Data Bank (PDB ID 9GLF, 1.4 Å, R_{work} = 14.7 and R_{free} = 18.1, **Table S5**).

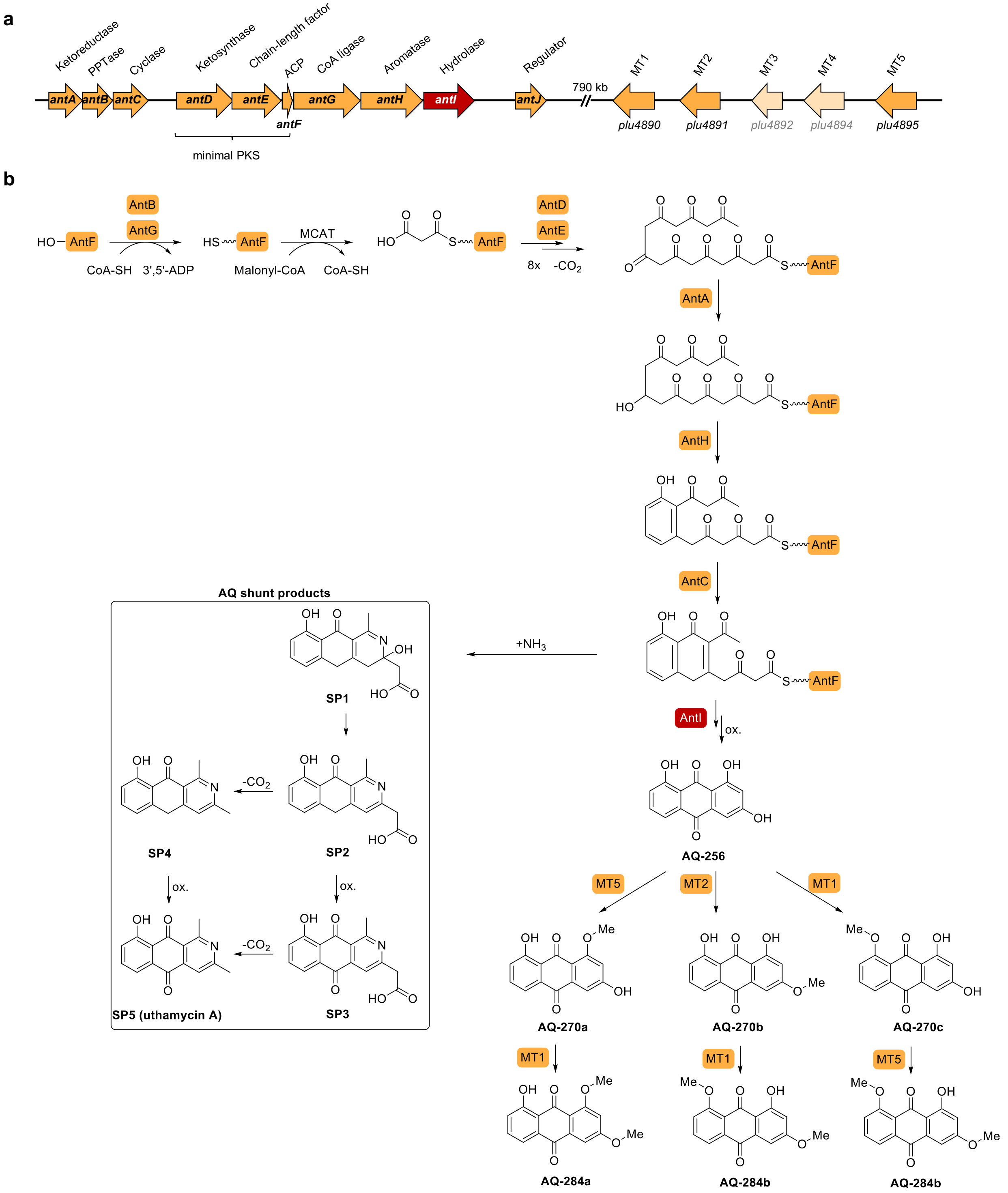

Figure S1. The *ant* (*antA–J*) biosynthetic gene cluster[5] and associated site-specific tailoring methyltransferases[1] involved in AQ production. The proposed biosynthetic pathway of AQs and AQ shunt products is shown and highlights AQ-270a and AQ-284a as the primary mono- and dimethylated AQ products in *P. laumondii* TTO1.

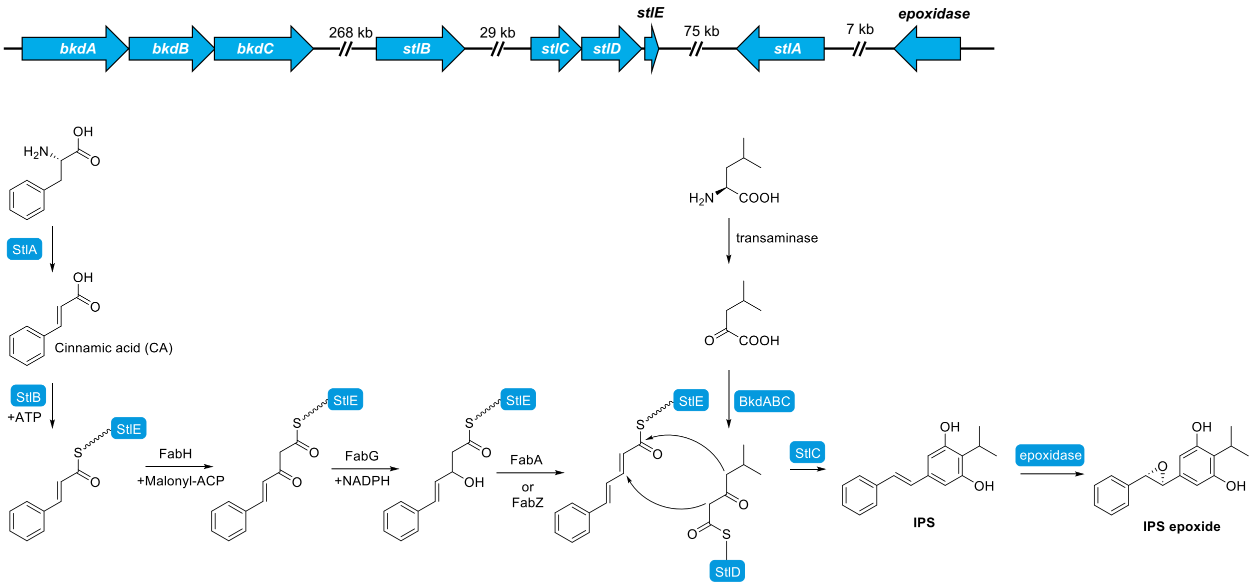

Figure S2. Biosynthetic gene cluster and the corresponding pathway of IPS formation in *P. laumondii* TTO1[12].

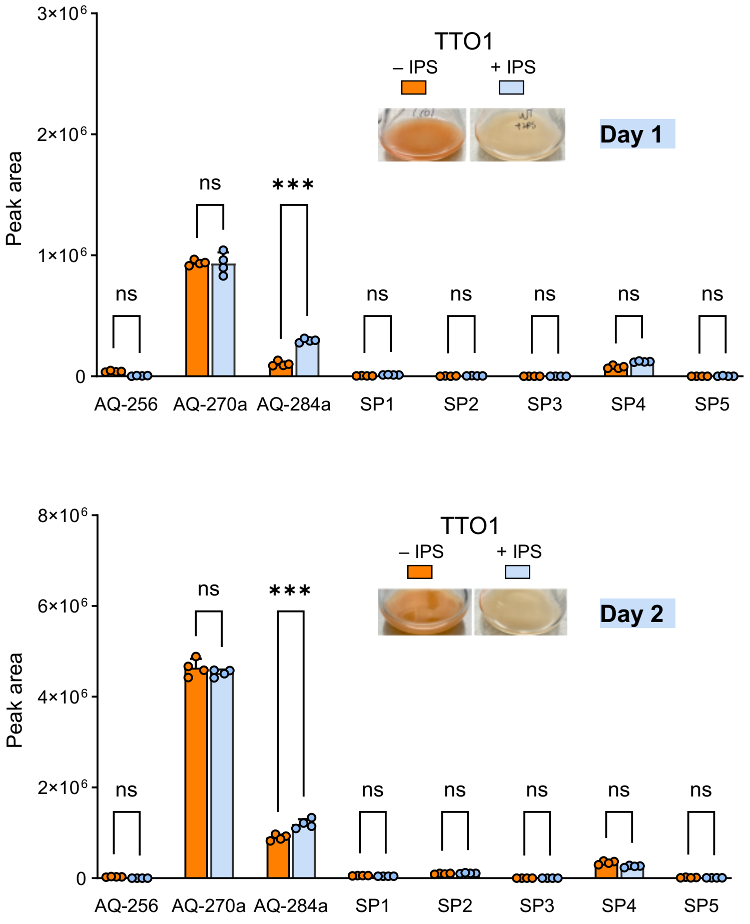

Figure S3. HR-LCMS analysis of AQ and AQ shunt product (SP1-SP5) production in TTO1 in the presence/absence of IPS after one and two days. EIC peak areas are shown in **Table S1**. Error bars represent mean ± s.d. of four replicates. Statistical significance was determined using Bonferroni's multiple comparisons test, with asterisks indicating significant differences between samples. (ns = not significant, * = P≤0.033, ** = P≤0.002, *** = P≤0.001).

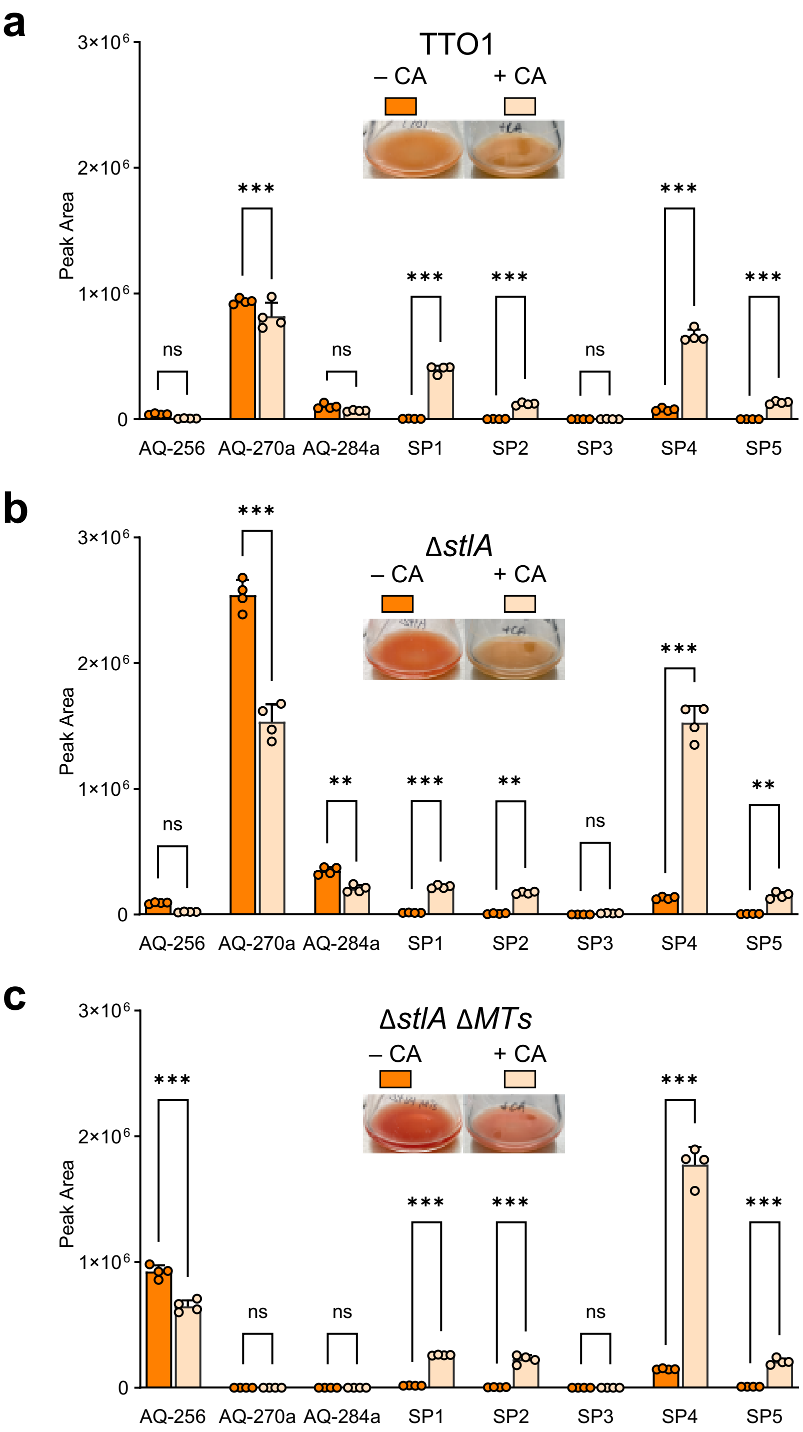

Figure S4. HR-LCMS analysis of the production of AQs and AQ shunt products SP1-SP5 in the absence/presence of fed CA after 1 day. EIC peak areas are shown in **Table S1**. Error bars indicate mean ± s.d. for four replicate samples. Statistical analysis was performed with the Bonferroni's multiple comparisons test. Asterisk show significant difference between CA-fed and non CA-fed samples (ns = not significant, * = P≤0.033, **= P≤0.002, *** = P≤0.001.).

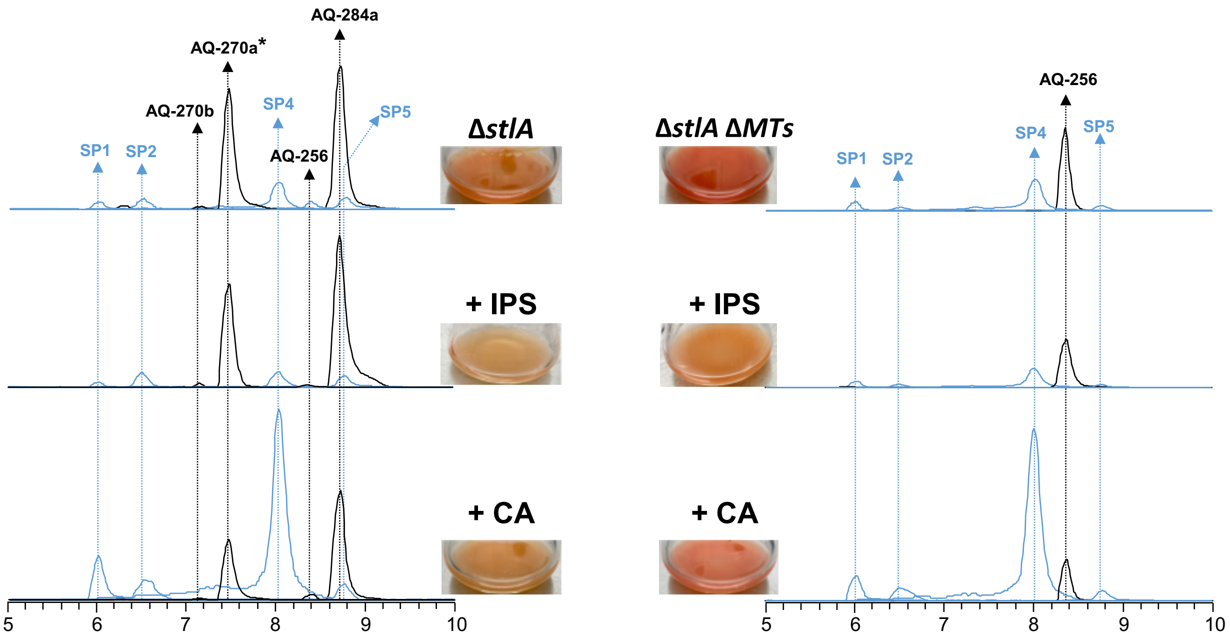

Figure S5. Extracted ion chromatograms (EICs) of AQs and AQ shunt products (SPs) from mutants Δ*stlA* and Δ*stlA* Δ*MTs* in different culture conditions after 2 days. Asterisk means peaks of AQ-270a were 5-fold decreased in order to fit it into the chromatogram.

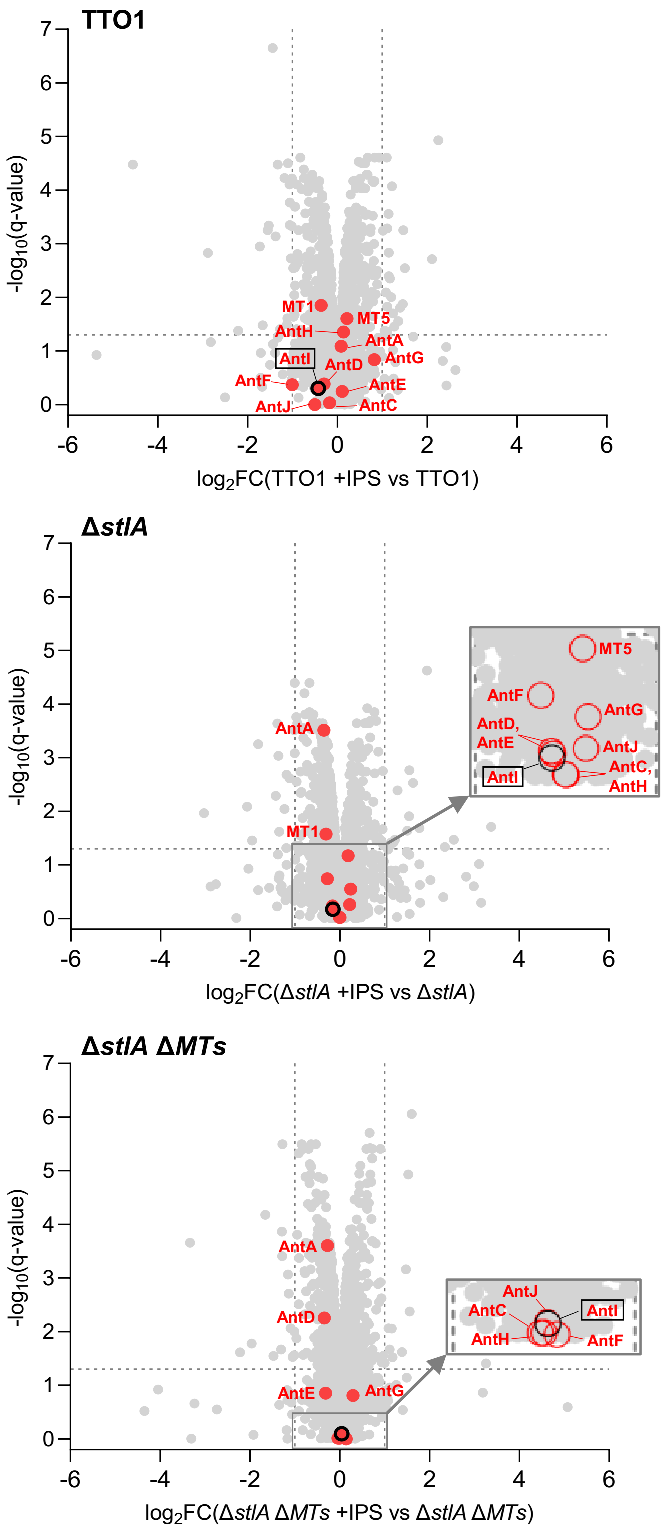

Figure S6. Volcano plot of the comparative proteomic analysis of TTO1, Δ*stlA*, Δ*stlA* Δ*MTs* in the presence and absence of IPS with detailed annotation of proteins (red dots) involved in AQ biosynthesis. Dashed lines show cutoff values q = 0.05, and FC (fold change) = 2.

Table S1. Qualification of AQs, AQ shunts, and IPS in different strains based on the integrated EIC (extracted ion chromatogram) peak areas. These $[M+H]^+$ *m/z* used for EIC peaks integration were 257.0444 (AQ-256), 271.0601 (AQ-270a), 285.0757 (AQ-284a), 302.1022 (SP1), 284.0917 (SP2), 298.0709 (SP3), 240.1019 (SP4), and 254.0811 (SP5).

Samples – day 1	AQ-256	AQ-270a	AQ-284a	SP1	SP2	SP3	SP4	SP5
TTO1-1d #1	34,741	966,763	132,771	7,022	3,081	0	92,257	0
TTO1-1d #2	37,181	932,276	90,748	2,660	1,623	0	68,412	0
TTO1-1d #3	41,877	915,035	88,896	4,062	1,349	0	67,689	0
TTO1-1d #4	47,543	941,462	100,214	5,160	3,349	0	81,092	0
TTO1-1d + CA #1	4,198	974,086	62,297	352,350	124,285	1,675	738,095	145,700
TTO1-1d + CA #2	5,599	769,060	74,124	414,350	109,230	1,532	632,632	137,700
TTO1-1d + CA #3	5,878	728,881	69,903	413,920	134,980	3,121	640,010	129,600
TTO1-1d + CA #4	9,910	810,446	68,228	411,900	120,275	1,696	646,311	120,690
TTO1-1d + IPS #1	0	830,815	294,599	11,968	6,259	0	117,114	1,864
TTO1-1d + IPS #2	4,362	898,675	298,508	11,183	4,583	0	122,253	2,171
TTO1-1d + IPS #3	6,137	962,676	278,227	12,496	4,010	0	120,375	1,087
TTO1-1d + IPS #4	5,749	1,042,111	314,493	14,027	3,348	0	128,211	6,292
Δ*stlA*-1d #1	83,529	2,516,270	372,165	14,433	4,385	0	143,265	4,460
Δ*stlA*-1d #2	93,408	2,388,168	316,004	15,547	7,628	0	123,827	6,332
Δ*stlA*-1d #3	96,831	2,584,119	378,647	17,164	12,484	0	140,293	5,842
Δ*stlA*-1d #4	99,633	2,679,154	330,988	15,017	12,005	0	123,365	6,243
Δ*stlA*-1d + CA #1	16,015	1,377,392	184,578	228,857	183,866	8,934	1,489,859	140,242
Δ*stlA*-1d + CA #2	21,197	1,472,438	182,046	212,951	165,912	14,745	1,351,009	125,246
Δ*stlA*-1d + CA #3	21,324	1,677,155	244,202	237,464	165,049	10,743	1,632,337	183,352
Δ*stlA*-1d + CA #4	24,555	1,618,646	213,369	208,148	183,132	8,414	1,634,373	165,761
Δ*stlA*-1d + IPS #1	9,667	1,765,312	529,493	22,427	16,023	1,357	200,217	10,488
Δ*stlA*-1d + IPS #2	10,514	2,378,925	873,899	29,469	25,941	3,168	239,507	3,741
Δ*stlA*-1d + IPS #3	11,890	2,017,494	662,393	24,109	19,297	2,049	209,760	7,879
Δ*stlA*-1d + IPS #4	15,488	1,908,245	583,786	20,430	15,927	1,794	189,557	9,409
Δ*stlA* Δ*MTs* -1d #1	860,257	0	0	18,641	4,574	0	145,895	9,117
Δ*stlA* Δ*MTs* -1d #2	925,266	0	0	17,645	4,238	0	144,836	8,411
Δ*stlA* Δ*MTs* -1d #3	929,000	0	0	16,123	7,151	0	155,286	8,464
Δ*stlA* Δ*MTs* -1d #4	981,809	0	0	17,933	6,021	0	148,560	9,355
Δ*stlA* Δ*MTs* -1d + CA #1	624,084	0	0	256,140	180,601	1,290	1,814,761	202,768
Δ*stlA* Δ*MTs* -1d + CA #2	666,128	0	0	258,985	244,384	1,032	1,895,431	207,503
Δ*stlA* Δ*MTs* -1d + CA #3	708,113	0	0	262,032	222,494	1,088	1,819,177	183,148
Δ*stlA* Δ*MTs* -1d + CA #4	600,048	0	0	264,623	260,384	2,506	1,566,845	242,954
Δ*stlA* Δ*MTs* -1d + IPS #1	763,455	0	0	26,768	7,514	0	190,608	13,737
Δ*stlA* Δ*MTs* -1d + IPS #2	701,572	0	0	29,098	8,834	0	191,884	14,771
Δ*stlA* Δ*MTs* -1d + IPS #3	687,520	0	0	28,988	5,292	0	214,927	9,155
Δ*stlA* Δ*MTs* -1d + IPS #4	683,346	0	0	31,033	8,386	0	215,842	11,494
**Samples – day 2**	**AQ-256**	**AQ-270a**	**AQ-284a**	**SP1**	**SP2**	**SP3**	**SP4**	**SP5**
TTO1-2d #1	30,802	4,889,676	972,880	58,342	106,805	0	371,871	24,471
TTO1-2d #2	32,018	4,668,494	932,318	59,263	94,044	0	385,001	16,140
TTO1-2d #3	28,003	4,586,084	825,840	55,377	91,223	0	335,595	13,602
TTO1-2d #4	35,556	4,423,427	867,837	57,734	109,743	0	300,491	11,955
TTO1-2d + CA #1	15,457	2,518,745	576,430	301,470	146,966	5,312	1,702,671	87,968
TTO1-2d + CA #2	19,400	2,498,035	543,817	290,145	144,848	5,121	1,488,542	74,422
TTO1-2d + CA #3	23,283	2,440,375	506,511	266,581	142,746	4,934	1,575,306	90,494
TTO1-2d + CA #4	26,759	2,615,921	465,872	269,521	136,519	6,169	1,516,942	72,893
TTO1-2d + IPS #1	2,272	4,583,594	1,334,842	45,948	107,273	0	270,110	12,889
TTO1-2d + IPS #2	3,958	4,576,535	1,149,035	45,328	109,205	0	283,258	11,262
TTO1-2d + IPS #3	2,669	4,508,526	1,094,869	43,956	103,842	0	231,860	12,986
TTO1-2d + IPS #4	3,064	4,414,438	1,218,201	48,046	124,397	0	263,771	12,535
Δ*stlA*-2d #1	45,052	7,114,276	1,628,569	74,295	147,099	0	488,920	34,342
Δ*stlA*-2d #2	42,555	6,514,414	1,366,561	74,673	142,838	0	444,083	30,988
Δ*stlA*-2d #3	22,587	7,164,996	1,517,324	79,306	161,157	0	491,231	44,663
Δ*stlA*-2d #4	30,755	7,198,126	1,512,170	78,553	165,240	0	456,770	36,175
Δ*stlA*-2d + CA #1	22,991	3,881,483	1,271,997	500,230	272,303	4,934	2,758,580	157,315
Δ*stlA*-2d + CA #2	18,902	3,970,374	1,294,629	428,512	273,615	4,547	2,398,264	120,987
Δ*stlA*-2d + CA #3	17,621	3,666,981	1,352,003	465,009	289,533	4,337	2,642,016	126,697
Δ*stlA*-2d + CA #4	19,180	4,102,357	1,344,973	457,901	284,760	4,214	2,638,398	140,780
Δ*stlA*-2d + IPS #1	8,508	5,561,808	1,366,131	56,388	183,041	5,168	339,900	28,854
Δ*stlA*-2d + IPS #2	6,450	5,319,567	1,473,639	58,459	180,434	6,413	356,901	29,126
Δ*stlA*-2d + IPS #3	6,327	5,431,392	1,411,135	55,177	174,837	4,923	324,627	21,132
Δ*stlA*-2d + IPS #4	12,034	5,645,705	1,476,094	62,744	196,986	7,091	346,251	20,540
Δ*stlA* Δ*MTs* -2d #1	1,118,755	0	0	89,649	38,641	0	548,980	50,846
Δ*stlA* Δ*MTs* -2d #2	1,167,196	0	0	83,721	36,713	0	464,884	32,314
Δ*stlA* Δ*MTs* -2d #3	1,238,104	0	0	83,003	42,201	0	478,909	36,586
Δ*stlA* Δ*MTs* -2d #4	1,257,333	0	0	91,557	38,449	0	511,076	30,899
Δ*stlA* Δ*MTs* -2d + CA #1	609,215	0	0	462,189	218,500	6,454	2,489,868	131,140
Δ*stlA* Δ*MTs* -2d + CA #2	607,344	0	0	492,778	222,366	7,183	2,614,499	138,337
Δ*stlA* Δ*MTs* -2d + CA #3	598,973	0	0	477,356	203,422	8,011	2,409,110	159,087
Δ*stlA* Δ*MTs* -2d + CA #4	645,879	0	0	460,976	195,723	6,038	2,569,208	145,437
Δ*stlA* Δ*MTs* -2d + IPS #1	660,565	0	0	57,930	24,599	0	364,757	23,172
Δ*stlA* Δ*MTs* -2d + IPS #2	679,045	0	0	60,436	13,528	0	339,485	24,963
Δ*stlA* Δ*MTs* -2d + IPS #3	692,034	0	0	61,956	22,717	0	370,529	31,131
Δ*stlA* Δ*MTs* -2d + IPS #4	669,231	0	0	67,181	26,509	0	402,950	32,439

Table S2. Strains used in this study.

Strain	Genotype/Description	Reference
E. coli		
DH10B	Cloning strain; F⁻ *mcr*A, Δ(*mrr-hsdRMS-mcrBC*) Φ80*lacZ* Δ*M15* Δ*lac*X74 *rec*A1 *end*A1,araD139, Δ(*ara-leu*)7697 *gal*U, *gal*K, *rps*L,*nup*G, λ-	Invitrogen
ST18	Conjugation strain: *E. coli* S17-1 *λpir* Δ*hem*A	Ref[13]
BL21 (DE3)	Protein expression strain: F– *ompT hsdSB (rB– mB–) gal dcm* λ(DE3)	Novagen
ST18 pAR20_ΔstlA	ST18 contains the gene *stlA* (*plu2234*) deletion plasmid pAR20_ΔstlA (Km^R)	This study
BL21 (DE3) ZQ40	AntI expression strain	Ref[5]
***Photorhabdus laumondii* TTO1**		
TTO1	*Photorhabdus laumondii* TTO1 (wild type)	DSM15139
Δ*MTs*	TTO1 with the deletion of *genes plu4890-4895 (namely PLUMV2_24550-24580)*	Ref[1]
Δ*stlA*	TTO1 with the deletion of gene *stlA* (*plu2234, namely PLUMV2_11885*)	Ref[2]
Δ*stlA* Δ*MTs*	TTO1 with the deletion of genes *stlA* and *plu4890-4895*	This study

Table S3. Plasmids used in this study.

Plasmid	Genotype/Description	Reference
pAR20	pSEVA231 *tet*R P_{tet}-λRed *araC* P_{BAD} codon optimized FnCas12a *sac*B crRNA framework	Ref[2]
pAR20_ΔstlA	Plasmid used for gene deletion of *stlA* by CRIPSR-Cfp1	Ref[2]
ZQ40	ColE1 ori, Km^R, T7*lac* promoter*, antI;* AntI expression plasmid	Ref[5]

Table S4. Primers used in this study.

Primer	Sequence	usage
LS144	GCGTGCCATCGTAGGTTTTATTCAC	Verification primers for the deletion of *stlA*
LS145	GATGTAACAACCTTTCAGCCCACGG	

Table S5. Crystallographic data collection and refinement statistics.

	AntI:CA
<u>**Crystal parameters**</u>	
Space group	$C222_1$
Cell constants	a= 54.4 Å
	b= 154.5 Å
	c= 91.1 Å
<u>**Data collection**</u>	
Beam line	X06SA, SLS
Wavelength (Å)	1.0
Resolution range (Å)[b]	30-1.4
	(1.5-1.4)
No. observations	335,698
No. unique reflections[c]	74,543
Completeness (%)[b]	98.4 (98.6)
R_{merge} (%)[b,d]	3.0 (66.2)
I/σ (I)[b]	20.2 (2.0)
<u>**Refinement (REFMAC5)**</u>	
Resolution range (Å)	30-1.4
No. refl. working set	70,809
No. refl. test set	3,727
No. non hydrogen	3,387
No. of ligand atoms	11
Solvent	295
R_{work}/R_{free} (%)[e]	14.7 / 18.1
r.m.s.d. bond (Å) / angle (°)[f]	0.006 / 1.4
Average B-factor (Å^2)	25.7
Ramachandran Plot (%)[g]	98.1 / 1.9 / 0
PDB accession code	9GLF

[a] Asymmetric unit
[b] The values in parentheses for resolution range, completeness, R_{merge} and I/σ (I) correspond to the highest resolution shell
[c] Data reduction was carried out with XDS and from a single crystal. Friedel pairs were treated as identical reflections
[d] $R_{merge}(I) = \Sigma_{hkl}\Sigma_j \mid I(hkl)_j - \langle I(hkl)\rangle \mid / \Sigma_{hkl} \Sigma_j I(hkl)_j$, where $I(hkl)_j$ is the jth measurement of the intensity of reflection hkl and <I(hkl)> is the average intensity
[e] $R = \Sigma_{hkl} \mid |F_{obs}| - |F_{calc}| \mid / \Sigma_{hkl} |Fobs|$, where R_{free} is calculated without a sigma cut off for a randomly chosen 5% of reflections, which were not used for structure refinement, and R_{work} is calculated for the remaining reflections
[f] Deviations from ideal bond lengths/angles
[g] Percentage of residues in favored region / allowed region / outlier region

Supplementary References

1. Huber, E. M.; Kreling, L.; Heinrich, A. K.; Dünnebacke, M.; Pöthig, A.; Bode, H. B.; Groll, M., A set of closely related methyltransferases for site-specific tailoring of anthraquinone pigments. *Structure* **2023,** *31* (5), 573-583.e5.
2. Rill, A.; Zhao, L.; Bode, H. B., Genetic toolbox for Photorhabdus and Xenorhabdus: pSEVA based heterologous expression systems and CRISPR/Cpf1 based genome editing for rapid natural product profiling. *Microb Cell Fact* **2024,** *23* (1), 98.
3. Hapeshi, A.; Benarroch, J. M.; Clarke, D. J.; Waterfield, N. R., Iso-propyl stilbene: a life cycle signal? *Microbiology (Reading)* **2019,** *165* (5), 516-526.
4. Neubacher, N.; Tobias, N. J.; Huber, M.; Cai, X.; Glatter, T.; Pidot, S. J.; Stinear, T. P.; Lütticke, A. L.; Papenfort, K.; Bode, H. B., Symbiosis, virulence and natural-product biosynthesis in entomopathogenic bacteria are regulated by a small RNA. *Nat Microbiol* **2020,** *5* (12), 1481-1489.
5. Zhou, Q.; Bräuer, A.; Adihou, H.; Schmalhofer, M.; Saura, P.; Grammbitter, G. L. C.; Kaila, V. R. I.; Groll, M.; Bode, H. B., Molecular mechanism of polyketide shortening in anthraquinone biosynthesis of Photorhabdus luminescens. *Chem Sci* **2019,** *10* (25), 6341-6349.
6. Kabsch, W., XDS. *Acta Crystallographica Section D* **2010,** *66* (2), 125-132.
7. Schuttelkopf, A. W.; van Aalten, D. M. F., PRODRG: a tool for high-throughput crystallography of protein-ligand complexes. *Acta Crystallographica Section D* **2004,** *60* (8), 1355-1363.
8. Murshudov, G. N.; Skubak, P.; Lebedev, A. A.; Pannu, N. S.; Steiner, R. A.; Nicholls, R. A.; Winn, M. D.; Long, F.; Vagin, A. A., REFMAC5 for the refinement of macromolecular crystal structures. *Acta Crystallographica Section D* **2011,** *67* (4), 355-367.
9. Emsley, P.; Lohkamp, B.; Scott, W. G.; Cowtan, K., Features and development of Coot. *Acta Crystallographica Section D* **2010,** *66* (4), 486-501.
10. Morris, R. J.; Perrakis, A.; Lamzin, V. S., ARP/wARP and automatic interpretation of protein electron density maps. *Methods Enzymol* **2003,** *374*, 229-44.
11. Williams, C. J.; Headd, J. J.; Moriarty, N. W.; Prisant, M. G.; Videau, L. L.; Deis, L. N.; Verma, V.; Keedy, D. A.; Hintze, B. J.; Chen, V. B.; Jain, S.; Lewis, S. M.; Arendall, W. B., 3rd; Snoeyink, J.; Adams, P. D.; Lovell, S. C.; Richardson, J. S.; Richardson, D. C., MolProbity: More and better reference data for improved all-atom structure validation. *Protein Sci* **2018,** *27* (1), 293-315.
12. Kavakli, S.; Grammbitter, G. L. C.; Bode, H. B., Biosynthesis of the multifunctional isopropylstilbene in *Photorhabdus laumondii* involves cross-talk between specialized and primary metabolism. *Tetrahedron* **2022,** *128*, 133116.
13. Thoma, S.; Schobert, M., An improved *Escherichia coli* donor strain for diparental mating. *FEMS Microbiol Lett* **2009,** *294* (2), 127-32.
